# Gene editing in cancer therapy: overcoming drug resistance and enhancing precision medicine

**DOI:** 10.1038/s41417-025-00959-9

**Published:** 2025-09-18

**Authors:** Hyeonjeong Park, Suyeun Yu, Taeyoung Koo

**Affiliations:** 1 Department of Regulatory Science, Graduate School, Kyung Hee University, 26 Kyungheedae-ro, Dongdaemun-gu, Seoul, 02447 Republic of Korea; 2 College of Pharmacy and Institute of Integrated Pharmaceutical Sciences, Kyung Hee University, 26 Kyungheedae-ro, Dongdaemun-gu, Seoul, 02447 Republic of Korea

**Keywords:** Targeted gene repair, Targeted therapies

## Abstract

The CRISPR system has revolutionized cancer gene therapy, offering unparalleled precision in genetic manipulation for targeted oncogene disruption, mutation correction, and immune system modulation. This breakthrough tool has demonstrated remarkable potential in overcoming drug resistance, enhancing chemotherapy sensitivity, and improving immunotherapy strategies such as CRISPR-engineered CAR-T cells. Additionally, oncolytic virus-mediated CRISPR delivery has emerged as a novel approach for tumor-specific gene editing, minimizing off-target effects. The rapid transition of CRISPR-based cancer therapeutics from preclinical research to clinical trials underscores its therapeutic potential. This review explores the latest advancements in CRISPR applications for cancer therapy, including gene knockout, base editing for mutation correction, and integration with immune and viral therapies. Despite significant progress, challenges such as off-target effects, immune responses, and delivery limitations remain key hurdles. We discuss current strategies to enhance CRISPR safety and efficacy, emphasizing its potential for personalized cancer treatment.

## Introduction

Conventional chemotherapy has long been the cornerstone of cancer treatment, employing a systemic approach to target rapidly dividing cancer cells. Despite its widespread application, chemotherapy is associated with significant limitations that undermine its efficacy and safety. A primary drawback is its inability to distinguish between cancer cells and rapidly dividing normal cells, such as those in the gastrointestinal tract, hair follicles, and bone marrow. This non-selectivity results in severe off-target effects, including anemia, nausea, hair loss, and immunosuppression, significantly compromising patient quality of life and limiting treatment tolerability [1]. Additionally, conventional chemotherapeutic agents primarily aim to temporarily suppress tumor growth without addressing the underlying genetic mutations or dysregulated pathways that drive oncogenesis. This limitation necessitates continuous or repeated treatment cycles, which exacerbate cumulative side effects and further promote drug resistance mechanisms. Prolonged drug exposure often induces secondary mutations in drug targets, leading to therapeutic failure and exacerbating cancer progression [2–5]. To address these challenges, innovative therapeutic modalities such as targeted therapies, immunotherapies, and personalized medicine have been developed. These approaches strive to enhance selectivity, minimize adverse effects, and improve clinical outcomes. Among these advancements, clustered regularly interspaced short palindromic repeat (CRISPR)/CRISPR-associated protein (Cas) technologies have emerged as a therapeutic platform, offering unprecedented precision in genome editing for cancer therapy.

Recent breakthroughs in CRISPR-based technologies, including base editing, prime editing, and epigenetic regulation, have expanded its therapeutic potential beyond simple gene knockout, enabling precise modification of single nucleotide variants (SNVs) and transcriptional control of oncogenes and tumor suppressors. Furthermore, its integration with immunotherapies, such as CRISPR-engineered Chimeric antigen receptor (CAR)-T cells, has demonstrated remarkable potential in enhancing antitumor responses and overcoming immune evasion mechanisms. This review explores the potential of CRISPR-based cancer therapeutics to address the limitations of conventional treatments, highlights recent advancements in CRISPR applications, and discusses their convergence with emerging precision medicine strategies. By examining both the opportunities and challenges of CRISPR in oncology, we provide a comprehensive perspective on its role in shaping the future of cancer therapy (Table 1).

**Table 1:** Summary of CRISPR engineering for cancer therapy.

Technology	Type	Gene	Mutation	Cancer	Reference
Cas	Oncogene	*KRAS*	G12S, G12D	NSCLC	(23, 69)
	Oncogene	*EGFR*	-	NSCLC	(24)
	Tumor suppressor	*DEPCD5*	DUF5803 domain	Hepatocellular carcinoma	(26)
	MDR	*ABCB1*	-	Colorectal cancer	(39)
	Oncogene	*MCL-1*	-	Breast cancer	(61)
	Immune-related	*CD274*	-	Colorectal cancer	(64)
	Tumor suppressor	*TGFBR2*		Pancreatic carcinoma	(62)
	Oncogene	*SOX2*	-	head and neck squamous cell carcinoma	(67)
Base editor	Oncogene	*EGFR*	T790M	NSCLC	(17)
	Tumor suppressor	*TP53*	R273H	NSCLC	(17)
	Oncogene	*KRAS*	G12D	Sarcoma	(28)
	Tumor suppressor	*TP53*	R175H	Colorectal cancer	(28)
	Tumor suppressor	*TERT*	-124C>T	Hepatocellular carcinoma	(29)
	Immune-related	*TRAC,CD7*, *CD52,PDCD1*	-	T-ALL	(56)
	Immune-related	*TRBC, CD52,CD7*	-	T-ALL	(57)
Prime editor	Oncogene	*KRAS*	G13D	Colon cancer, pancreatic cancer	(33)

Non-small cell lung cancer, NSCLC; Acute lymphoblastic leukemia, ALL; MDR, multidrug resistance

## Comparative analysis of CRISPR nucleases, base editing, and prime editing tools in cancer therapy

**Fig. 1:**
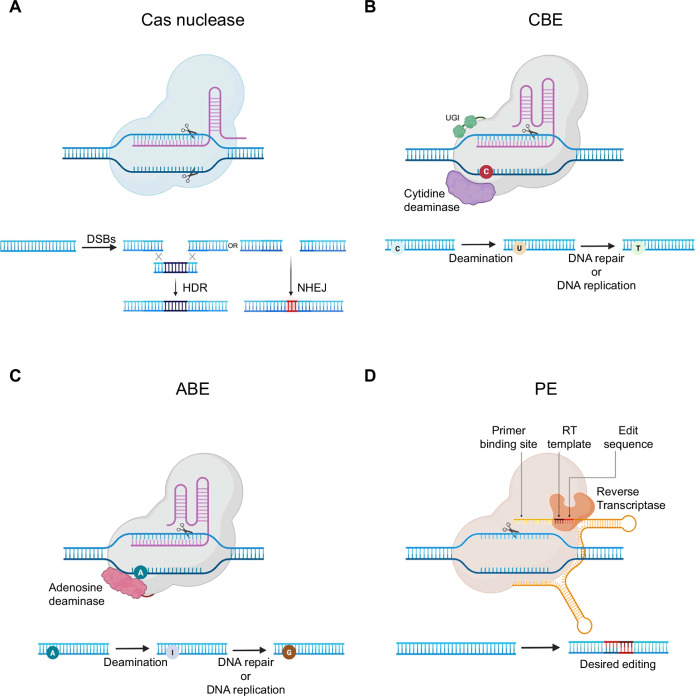
The CRISPR/Cas tools. **A** Cas nucleases introduce double-strand breaks (DSBs) in the genome, which are repaired via homology-directed repair (HDR) or non-homologous end joining (NHEJ). **B** Cytidine base editors (CBEs), composed of cytidine deaminase and uracil glycosylase inhibitor (UGI), deaminate cytosine (C), ultimately converting it into thymine (T). **C** Adenine base editors (ABEs) utilize adenosine deaminase to convert adenine (A) into inosine (I), which is interpreted as guanine (G) during DNA repair or replication. **D** Prime editors (PEs) consist of reverse transcriptase, nCas9, and a prime editing guide RNA (pegRNA) containing a primer binding site, reverse transcriptase template, and edit sequence. The pegRNA binds to the target DNA strand, enabling the precise modifications.

The CRISPR system, originally discovered as an adaptive immune mechanism in bacteria and archaea for viral defense, has revolutionized genome editing technologies [6–8]. At its core, the CRISPR system employs Cas endonucleases, which recognize specific protospacer adjacent motifs (PAMs) and generate double-strand breaks (DSBs) in the genome [9, 10]. This process is guided by RNA molecules, either as a combination of CRISPR RNA and *trans*-activating CRISPR RNA or a chimeric single-guide RNA (sgRNA) [11, 12]. Following DSB induction, endogenous DNA repair pathways, non-homologous end joining (NHEJ) or homology-directed repair (HDR), mediate the resolution of DNA breaks, thereby enabling genomic modifications in multicellular organisms (Fig. 1A) [13]. Although this approach enables targeted gene knockout, its clinical application in cancer gene therapy remains limited due to off-target effects and the risk of chromosomal translocations. Unintended DSBs at non-target loci may disrupt critical gene functions or lead to aberrant activation of proto-oncogenes, ultimately raising substantial safety concerns in therapeutic applications. Moreover, it remains inefficient for achieving precise genomic edits in cancer cells with complex oncogenic mutation profiles [14].

To address these limitations, CRISPR technology has evolved to include base editors (BEs) and prime editors (PEs), offering alternative strategies for more precise and safer genome modifications. BEs, including cytosine base editors (CBEs) and adenine base editors (ABEs) enable the conversion of cytosine-guanine (C:G) to thymine-adenine (T:A) and A:T to G:C base pairs, respectively, without DSBs [15, 16] (Fig. 1B, C). These systems allow precise base editing at targeted mutant alleles with reduced risk of indel formation, making them especially suitable for correcting oncogenic point mutations [17–20]. However, BEs are restricted to specific base conversions within a limited editing window and are not capable of performing transversions, insertions, or deletions [14].

PEs represent a more versatile genome editing technology that combines a modified Cas9 nickase with a reverse transcriptase and a prime editing guide RNA (pegRNA). This system enables the introduction of all types of point mutations, as well as insertions and deletions, without the need for DSBs or donor DNA templates (Fig. 1D) [14]. PEs expand the therapeutic scope of genome editing, particularly for complex genetic alterations. Nevertheless, PEs suffer from relatively low efficiency, delivery challenges due to its large component size, and a less mature development pipeline compared with Cas9 or BE platforms [21]. Although still undergoing optimization, engineering PE systems have significantly improved their efficiency. These cutting-edge CRISPR-based approaches hold significant potential in treating cancers at the molecular level. Despite recent improvements in CRISPR systems for gene editing in cancer therapies, their overall efficiency remains limited by factors such as the accessibility of the target site and the delivery method. Research aimed at engineering Cas variants, optimizing guide RNA structure, and developing high-throughput screening tools has improved editing performance while minimizing off-target effects.

## Gene knockout to inactivate oncogenes

**Fig. 2:**
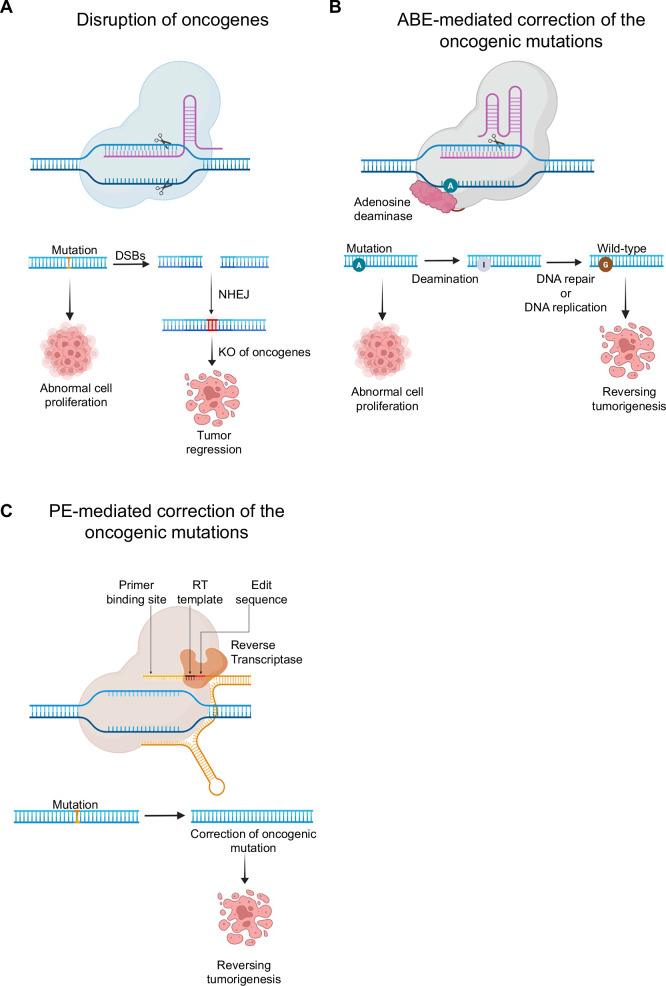
Applications of CRISPR technology in cancer therapy. **A** The Cas nucleases enable the precise knockout of mutated oncogenes that drive abnormal cell proliferation in cancer cells, leading to tumor regression. **B** ABEs and **C** PEs offer enhanced precision in correcting oncogenic mutations in cancers, enabling targeted therapeutic interventions to reverse tumorigenesis.

Overexpression of oncogenes is a hallmark of various cancers, driving tumor progression and malignancy [22]. CRISPR-Cas9 has been utilized to target and disrupt oncogenes in cancers (Fig. 2A). For example, intratumoral administration of an adenoviral vector encoding SpCas9 and sgRNA targeting the *KRAS* G12S mutation markedly suppressed tumor growth in A549 lung cancer xenograft model [23]. Treated mice exhibited a 46% decrease in tumor volume compared to mock-treated controls. Another promising approach involved targeting *EGFR* using CRISPR-Cas12a delivered via an oncolytic adenoviral vector (oAd). Treatment with oAd-Cas12a targeting *EGFR* effectively knocked out approximately 25.8% of *EGFR* sequences in lung cancer xenografts, with no detectable off-target effects [24]. This resulted in an 88.6% reduction in tumor volume, demonstrating the potential of targeted gene disruption in vivo.

Beyond direct therapeutic application, CRISPR knockout strategies are also valuable for functional validation of potential therapeutic targets [25]. For example, CRISPR-Cas9-mediated knockout of *DEPDC5* revealed its critical role in regulating oxidative stress responses in hepatocellular carcinoma (HCC) cells. *DEPDC5*-deficient HCC cells exhibited increased viability under leucine-starvation conditions due to enhanced resistance to reactive oxygen species mediated by autophagy deficiency, as evidenced by decreased LC3-II levels and p62 accumulation. Clinically, low DEPDC5 expression in HCC patient samples correlated with elevated p62 accumulation and was associated with worse progression-free and overall survival, which were significantly worse in DEPDC5-negative cases compared to DEPDC5-positive cases, suggesting that DEPDC5 functions as a tumor suppressor under nutrient-deprived conditions [26].

## Genome editing for precise mutation correction using BEs and PEs

Genome instability and SNVs are critical drivers of cancer development, contributing to tumor initiation and progression [27]. Recent advancements in CRISPR technologies offer unprecedented precision in correcting oncogenic SNVs, potentially reversing tumorigenesis and restoring normal gene function. BEs have emerged as a promising platform for targeting critical oncogenic mutations (Fig. 2B). For instance, BEs have been successfully applied in patient-derived organoid (PDO) models targeting mutations in *KRAS* and *TP53*. In PDOs of pancreatic ductal adenocarcinoma, efficient correction of the *KRAS* G12D was achieved, with an editing frequency of 30%. Similarly, in PDOs of colorectal carcinoma, the *TP53* R175H mutation was corrected with an editing frequency of 70%. These precise corrections led to a marked depletion of mutant cells and significant suppression of abnormal growth in cancer organoids. These findings underscore the potential of the BE system to correct oncogenic mutations and support the development of personalized, highly effective cancer treatment [28]. In another study, the dead *Campylobacter jejuni* Cas9 fused with an ABE achieved nearly 100% correction of −124C>T mutation in the *TERT* promoter, a genetic alteration found in various cancers, including human HCC. This precise correction suppressed abnormal *TERT* promoter activity, inhibited telomerase function, and significantly reduced tumor growth in the liver [29].

While these studies highlight the therapeutic potential of CRISPR-mediated base editing approaches, a key limitation lies in the editing accessibility of oncogenic mutations. A comprehensive study assessed 31,555 SNV mutations across 20 cancer driver genes to evaluate their suitability for editing using three commonly used nucleases: SpCas9, SaCas9, and LbCpf1. The results showed that approximately half of these mutations could be targeted by at least one nuclease, with editing rates ranging from 20.7 to 70.7% depending on the genes [23]. Despite the encouraging coverage, this also underscores a major limitation in clinical cancer gene editing. Many pathogenic mutations remain untargetable due to the absence of compatible PAMs. This bottleneck underscores the need for continued innovation. To address this limitation, significant efforts have focused on expanding the targetable genomic landscape by engineering Cas variants with relaxed or alternative PAM specificities [9, 10, 30–32]. These advances have broadened the range of genomic loci accessible for editing, thereby increasing the potential to target a wider spectrum of oncogenic mutations in clinical applications.

Emerging genome editing tools such as PEs have shown potential to overcome some of these targeting limitations. For instance, PE corrected 12 *KRAS* mutations, encompassing 94% of all characterized *KRAS* variants, with editing efficiencies of up to 54.8% in HEK293T/17 cells (Fig. 2C). Notably, the PE corrected the *KRAS G13D* mutation to its wild-type sequence with editing efficiency of 36.1% in HCT116 human colon cancer cells and 18.7% in ASPC-1 pancreatic cancer cells, while minimizing the occurrence of unintended indel formations [33]. These advancements in CRISPR-based genome editing highlight the transformative potential of correcting oncogenic SNVs, paving the way for precise and personalized cancer therapies that address the underlying genetic drivers of malignancy.

## Overcoming drug resistance with CRISPR system

Despite significant advances in chemotherapy, both intrinsic and acquired drug resistance remains a major obstacle to successful cancer treatment. A wide range of mechanisms contributing to drug resistance include tumor heterogeneity, drug inactivation, targeted protein alteration, activation of the DNA repair system, and inhibition of cell death [34, 35]. Multidrug resistance (MDR) is a major challenge in cancer therapy, often caused by the overexpression of ATP-binding cassette (ABC) transporters, which actively efflux chemotherapeutic drugs out of cancer cells, reducing intracellular drug accumulation and diminishing treatment efficacy. For example, the overexpression of P-glycoprotein encoded by the *ABCB1*, a transmembrane transporter, has been shown to prevent the accumulation of chemotherapeutic agents such as doxorubicin and vincristine in cancer cells. This mechanism ultimately contributes to chemotherapy failure in colorectal cancer [36, 37]. Although P-glycoprotein inhibitors have been developed, their clinical application remains limited due to unfavorable pharmacokinetics and adverse side effects [38].

**Fig. 3:**
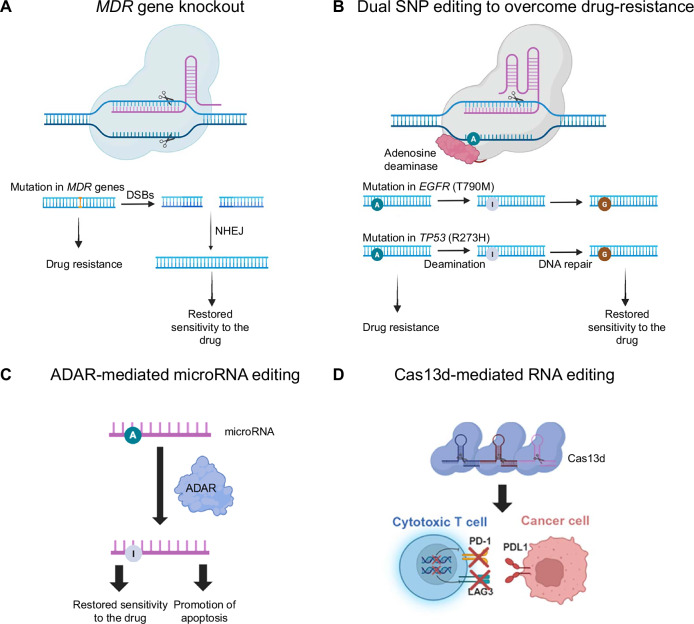
Schematic diagram of strategies to overcome the limitations of conventional cancer treatments. **A** Knocking out *multidrug resistance* (*MDR*) genes using Cas nucleases to combat drug resistance in cancer therapy. **B** The ABE offers a targeted approach for correcting oncogenic single nucleotide polymorphisms (SNPs), such as *EGFR* T790M and *TP53* R273H mutations, by converting adenine (A) to guanine (G) at specific loci, thereby restoring drug sensitivity and improving therapeutic outcomes. **C** Adenosine Deaminases Acting on RNA (ADAR)-mediated adenosine (A)-to-inosine (I) RNA editing enables transient suppression of oncogenic transcripts without permanent genomic modification. **D** Cas13d was utilized for multiplexed RNA knockdown to enhance CAR-T cell efficacy by suppressing the inhibitory immune checkpoint receptors PD-1 and LAG-3.

The CRISPR-Cas system provides a powerful approach to counteract MDR by targeting key molecular regulators involved in this process. Recently, the use of SpCas9 to knockout *ABCB1* in multidrug-resistant colorectal cancer cells (SW620/Ad300 and HCT-15) has demonstrated restored sensitivity to the *ABCB1* substrate drug, doxorubicin (Fig. 3A). This strategy enhanced intracellular accumulation of doxorubicin and altered tumor spheroid architecture, providing valuable insights into drug resistance mechanisms and potential therapeutic approaches [39].

Another innovative strategy to overcome drug resistance involves correcting drug resistance-inducing mutations using an ABE system. Pathogenic SNPs, such as *EGFR* T790M and *TP53* R273H mutations, are key contributors to gefitinib resistance. Targeting these SNPs for correction to the wild-type sequence presents a promising therapeutic avenue (Fig. 3B). This study demonstrated the efficacy of co-delivering an adenoviral vector encoding an ABE and guide RNAs targeting *EGFR* T790M and *TP53* R273H in gefitinib-resistant H1975 non-small cell lung cancer (NSCLC) harboring these mutations. This dual base editing approach achieved editing frequencies of 6.1% in *TP53* and 27.1% in *EGFR* in a xenograft mouse model of NSCLC, enhancing sensitivity to gefitinib and leading to successful tumor regression. These findings highlight the potential of ABE-mediated dual oncogenic SNP correction as a promising strategy for overcoming drug resistance in cancers [17].

## RNA editing in cancer therapy

The future of genome editing in cancer therapy may involve RNA editing systems [40]. RNA editing system provides a transient, reversible approach for suppressing oncogenic transcripts without altering the genome. Among these tools, adenosine deaminases acting on RNA (ADAR) enzymes catalyze the conversion of adenosine (A) to inosine (I) in RNA molecules, a process known as A-to-I RNA editing (Fig. 3C) [41, 42]. Leveraging this mechanism, the study was designed to overcome tyrosine kinase inhibitor (TKI) resistance in NSCLC by targeting miR-411–5p [42]. Indeed, A-to-I edited miR-411–5p directly suppressed MET and the ERK/MAPK pathway, leading to enhanced sensitivity to gefitinib and osimertinib in TKI-resistant NSCLC cell lines. In addition, intratumoral administration of RNA edited miR-411–5p reduced MET levels, suppressed tumor growth, and promoted apoptosis in NSCLC xenografts. These findings highlight ADAR-mediated RNA editing as a promising therapeutic strategy for modulating drug resistance dynamically, offering a non-permanent, precision oncology approach that avoids the risks associated with permanent genomic alterations.

Furthermore, CRISPR-Cas13 has been extensively utilized for RNA editing [43]. Building on this system, Cas13d was employed to enhance CAR-T cell therapy by multiplexed RNA knockdown of key genes regulating T-cell exhaustion, metabolism, and immune suppression (Fig. 3D) [44]. By using multiplexed effector guide arrays, a system for programmable RNA knockdown, the researchers achieved precise, reversible gene silencing without DNA modifications, addressing key limitations of conventional CRISPR-Cas9 editing. These findings highlight Cas13d-based transcriptomic engineering as a promising, non-permanent, precision approach for optimizing CAR-T cell therapy.

## Identifying critical target genes through CRISPR-based functional screening

**Fig. 4:**
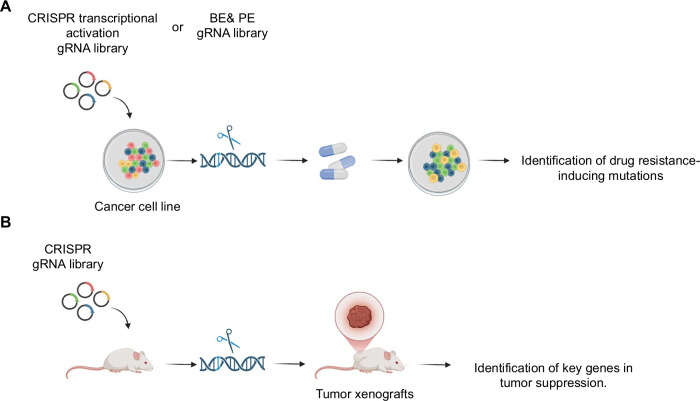
CRISPR-based genome-wide screening. CRISPR gRNA libraries were employed to **A** identify key mutations driving drug resistance and to **B** discover critical tumor suppressor genes.

High-throughput gRNA libraries are critical tool in CRISPR-based screening systems, driving significant advancements in understanding drug resistance mechanisms, facilitating drug discovery, and enabling personalized cancer therapies [45]. For example, a genome-scale CRISPR transcriptional activation library identified *LRP8* as a novel therapeutic target to overcome sorafenib resistance in HCC cells (Fig. 4A). Overexpression of *LRP8* suppresses apoptosis in HCC cells by upregulating β-catenin expression, significantly enhancing resistance to sorafenib [46]. Another study employed pooled guide RNA libraries for base editing and prime editing to investigate drug resistance mechanisms in *EGFR*. Their findings revealed that the T790A/Q791R mutations confer greater resistance to osimertinib compared to gefitinib (Fig. 4A). These insights offer valuable guidance for future clinical decision-making and personalized treatment strategies [47].

To better mimic human cancer progression, in vivo CRISPR screening models have been developed. Using a 67,405 gRNA library, genome-wide screening in a mouse model identified genes associated with lung cancer growth and metastasis (Fig. 4B). The top-scoring pro-apoptotic genes, *BID, PTEN*, and *CDKN2A*, were identified as critical players in tumor suppression [48].

## Clinical CRISPR trials in cancer therapy

CAR-T cell therapies have revolutionized cancer therapy by engineering T cells to specifically target tumor antigens. It utilizes synthetic antigen-recognizing receptors to enhance antitumor efficacy and mitigate immune suppression within the tumor microenvironment (TME) [49, 50]. From 2017 to 2024, the US Food and Drug Administration (FDA) has approved eight CAR-T cell therapies for cancer treatment, primarily targeting hematological malignancies such as lymphomas, leukemia, and multiple myeloma (Table 2) [51–53]. Despite their success in hematologic malignancies, challenges such as immune rejection, graft-versus-host disease (GvHD), and limited efficacy against solid tumors remain significant barriers [54]. Progress in CAR-T immunotherapy can be achieved by engineering cells to overcome two major challenges: mitigating GvHD, where donor-derived CAR-T cells attack the patient’s healthy tissues, and preventing the host-versus-graft response (HvGR), where the patient’s immune system attacks the infused CAR-T cells [55].

**Table 2:** Clinical CRISPR trials in cancer therapy.

Clinical Trial	Target Antigen	Cancer Type	Target Gene	Outcome
NCT04502446	CD70	T-cell lymphoma	*TRAC* and *B2M* knockout	47% ORR, 20% CR
NCT04227015	CD19,CD22	B-ALL	*CD52* and *TRAC* knockout	Enhanced activation, reduced relapse rates
NCT03399448	NY-ESO-1	Sarcoma, myeloma	*TRAC, TRBC,* and *PDCD1* knockout	Improved persistence and tumor targeting
NCT04037566	CD19	ALL, lymphoma	*MAP4K1* knockout	Increased response rate and T cell durability
NCT04171843	BCMA	Multiple myeloma	*TRAC* knockout	Favorable response in early-phase trials
NCT05885464	CD7	T-ALL	*TRAC,CD52,CD7*, and *PDCD1* base editing	Incomplete and complete remission
NCT05397184	CD7	T-ALL	*CD52, CD7*, and *TRBC* base editing	CR

*CR* complete remission, *ORR* overall response rate, *B-ALL* B-cell acute lymphoblastic leukemia, *T-ALL* T-cell acute lymphoblastic leukemia.

**Fig. 5:**
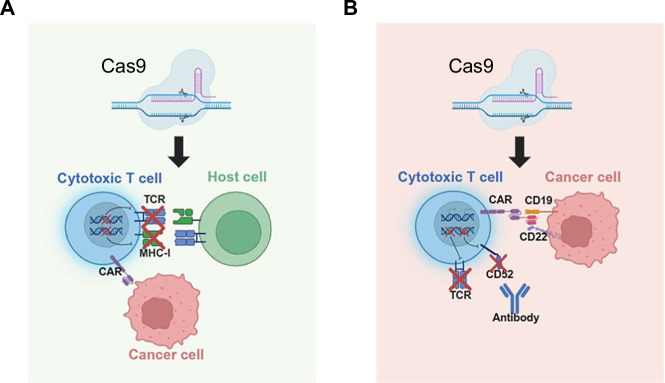
The CRISPR-engineered CAR-T therapy. **A** CRISPR-based strategies to prevent GvHD include suppressing TCR and MHC-I expression to eliminate the alloreactive potential of donor T-cells. **B** CRISPR-mediated silencing of CD52 and TCR in CAR-T cells confers resistance to HvGR and prevents GvHD, respectively.

The integration of CRISPR-Cas9 technology into CAR-T cell engineering has provided a novel approach to overcome these limitations, enhancing T cell functionality, persistence, and tumor specificity. Several clinical trials have been conducted to explore these advancements, as summarized in Table 2. The clinical trial NCT04502446 has been a milestone in CRISPR-based CAR-T therapy. This study evaluated CTX130, an allogeneic CRISPR-edited CAR-T cell therapy for patients with relapsed/refractory T-cell malignancies. In this trial, CRISPR-Cas9 was used to knock out the endogenous *TRAC* and *B2M* to prevent GvHD and immune rejection (Fig. 5A). The results showed an overall response rate (ORR) of 47%, with 20% of patients achieving complete remission, demonstrating the feasibility of CRISPR-engineered CAR-T therapies [56]. Another significant trial, NCT04227015, focuses on CRISPR-edited CAR-T cells for B-cell acute lymphoblastic leukemia (B-ALL). In this study, CRISPR-Cas9-mediated knockout of the *CD52* and *TRAC* genes in CAR-T cells provides resistance to HvGR and prevents GvHD, respectively. The early-phase data indicated enhanced T cell activation and reduced relapse rates (Fig. 5B) [57]. These trials highlight the potential of CRISPR in optimizing CAR-T cell therapy by improving persistence, reducing immune rejection, and enhancing antitumor efficacy. While early data are promising, further clinical validation is required to assess long-term safety, durability, and efficacy in both hematologic and solid tumors.

In addition to conventional CRISPR-Cas9-based editing, base editing has recently emerged as a next-generation therapy to improve the precision and safety of allogenic CAR-T therapies. BEAM-201 (NCT05885464) is an allogeneic, CD7-directed CAR-T cell therapy developed for treating CD7-positive T-cell malignancies such as T-cell acute lymphoblastic leukemia (T-ALL). Using CBE, this therapy introduces four targeted edits in *TRAC*, *CD7*, *CD52*, and *PDCD1*, thereby preventing GvHD, fratricide, immune rejection, and PD-L1-mediated immune evasion. In an initial clinical study (*n* = 3), no GvHD or neurotoxicity was observed. One patient experienced disease progression unrelated to treatment, while the remaining two achieved incomplete and complete responses, respectively, and proceeded to hematopoietic stem cell transplantation (HSCT) [58]. Similarly, BE-CAR7 (NCT05397184) is a base-edited CD7-targeted CAR-T cell therapy developed for T-ALL. By inactivating *CD52*, *CD7*, and the *TRBC* using CBE, this approach prevents fratricide, avoids GvHD, and eliminates the need for lymphodepleting serotherapy. In a Phase 1 trial involving three pediatric patients, one patient achieved complete remission and successfully underwent a second HSCT, another also proceeded to HSCT in remission, and one patient succumbed to a fungal infection. These results indicate the clinical potential of base-edited CAR-T therapies, while highlighting the importance of vigilant infection [59].

## Overcoming barriers in CAR-T cell therapy for solid tumors

CAR-T cell therapies have shown remarkable success in treating hematological malignancies. However, their efficacy in solid tumors remains limited due to challenges posed by the TME. To enhance CAR-T function under these hostile conditions, integration with CRISPR-based gene editing strategies has opened new possibilities for improving persistence, potency, and specificity [60]. One promising approach involves dual-targeting CAR-T cells recognizing both cancer cells and cancer-associated fibroblasts (CAFs). By simultaneously targeting CAFs, key mediators of ECM deposition and immunosuppression, this strategy improves T cell infiltration and antitumor activity in solid cancers. For example, CRISPR-Cas9-mediated knockout of *MCL-1* in breast cancer cells (T47D) significantly reduced CAF populations, supporting the potential of genome editing to modulate the TME and enhance therapeutic outcomes [61]. Similarly, targeting immunosuppressive pathways such as TGF-β signaling has demonstrated potential in enhancing CAR-T cell efficiency against solid tumors. CRISPR-Cas9-mediated knockout of *TGFBR2* in CAR-T cells reduced Treg induction and cellular exhaustion. Notably, TGFBR2-deficient CAR-T cells completely eradicated tumors in NPG xenograft models, highlighting the therapeutic potential of this strategy for improving CAR-T cell performance in solid tumor settings [62].

## Delivery of CRISPR components in cancer therapy: adenoviral vector-mediated CRISPR delivery

The integration of adenoviral vectors (Ad) with CRISPR technology represents a novel strategy for achieving cancer-specific gene therapy. Particularly, oncolytic Ad (oAd) selectively replicates within and lyses cancer cells while sparing normal tissues, making them an attractive vehicle for precise gene editing. Since the approval of Imlygic, the first oAd, in 2015, the number of clinical trials utilizing oAd has increased rapidly, underscoring their potential as a promising cancer treatment strategy [63]. By leveraging the tumor-specific replication properties of oAd, CRISPR can be effectively delivered into cancer cells, enabling targeted gene disruption with minimal off-target effects.

Recent studies have demonstrated the cancer-specific potential of CRISPR-Cas12a mediated by oAd to disrupt oncogenic signaling pathways. For instance, a single injection of oAd/Cas12a targeting *EGFR* led to an 88.6% reduction in tumor volume in A549 xenograft mice, highlighting the high efficacy of oAd combined with CRISPR-Cas12a in tumor suppression [24]. Similarly, an engineered an oAd to knockout *CD274* using the CRISPR-Cas9 and cloaked it with cell membranes displaying T cell-specific antigens, generating an oncolytic virus-T cell chimera. In a melanoma mouse model, this strategy led to significant tumor-specific accumulation of oAd, a 50% reduction in PD-L1 expression, and an 80% survival over 70 days following a single-dose administration [64]. These findings underscore the potential of combining oAd with the CRISPR system for precise, tumor-targeted therapy.

Although oAds have demonstrated promising outcomes in delivering CRISPR components for cancer therapy, several limitations hinder their full therapeutic potential. One key issue is the limited intratumoral retention of viral particles, which reduces sustained genome editing efficacy. Additionally, heterogeneity among tumor cells contributes to variable infection efficiency, resulting in inconsistent therapeutic effects [65]. Furthermore, oAds are susceptible to rapid clearance by the host immune responses. oAds that fail to internalize into tumor cells may be released into the bloodstream, where they are sequestered nonspecifically in normal tissues. This systemic exposure increases the likelihood of recognition by immune surveillance mechanisms, triggering strong antiviral immune responses that accelerate viral clearance and diminish therapeutic efficacy. These challenges underscore the need for enhanced targeting strategies, such as the use of tumor-specific promoters (e.g., hTERT) that are transcriptionally active predominantly in malignant cells, thereby confining Cas expression to cancerous tissues. In addition, the adaptation of microRNA-responsive regulatory elements enables selective activation of the CRISPR system, while capsid-modified Ad can enhance tumor tropism through targeted binding to cancer-specific surface markers, thereby improving the precision of CRISPR delivery in cancer therapy [66].

## Non-viral vector-mediated CRISPR delivery

Non-viral delivery, such as lipid nanoparticles (LNPs) and exosomes, offers alternatives to viral vectors due to their lower immunogenicity. In a preclinical study utilizing a xenograft mouse model of head and neck squamous cell carcinoma, co-encapsulation of Cas9 mRNA and sgRNA targeting *SOX2* within LNPs enabled efficient intratumoral delivery of a CRISPR system, achieving a 90% reduction in tumor growth and prolonged survival by 90% over a period of 84 days. Furthermore, complete tumor regression was observed in 50% of the treated mice, demonstrating the therapeutic potential of a tumor-specific CRISPR delivery platform [67]. However, the clinical efficacy of LNPs remains limited by rapid degradation of CRISPR components in circulation and suboptimal targeting efficiency, underscoring the need for further innovation in delivery technologies [68].

Exosomes, nanosized extracellular vesicles secreted by cells, have also been explored as natural carriers of CRISPR components. Exosome-mediated delivery of CRISPR-Cas9 plasmids targeting *KRAS-*G12D in pancreatic cancer cells achieved 58% gene knockdown. This was accompanied by significant inhibition of tumor proliferation both in vitro and in xenograft models [69]. However, to fully realize the therapeutic potential of this approach, further research is needed to improve CRISPR-Cas9 delivery efficiency and overcome barriers such as cellular uptake and nuclear entry [70].

## Challenges and future directions

CRISPR-based therapies hold tremendous potential for gene therapy, offering the potential to precisely manipulate genomes. A noble milestone in the field is the FDA approval of Casgevy, the first CRISPR-based cell therapy, which employs CRISPR-Cas9 to edit *BCL11A* in hematopoietic stem cells for treating hematologic disorders. This approval highlights the growing clinical relevance of CRISPR technologies. Emerging CRISPR-CAR-T therapies and utilization of oncolytic viruses are reshaping the immunotherapeutic landscape, enhancing tumor-specific targeting while reducing immune evasion. Despite its promise, several critical challenges must be addressed for successful clinical translation. One major challenge is the risk of off-target effects, which can lead to unintended mutations in non-cancerous cells. To address this risk, tumor-specific delivery platforms, engineered high-fidelity CRISPR variants, and real-time off-target detection technologies have been developed.

Another major hurdle is the tumor heterogeneity frequently observed in cancers. Variability in genetic and epigenetic landscapes across tumor cells can result in incomplete targeting, thereby compromising therapeutic efficacy. Emerging approaches such as multiplexed genome editing may provide solutions to overcome this complexity [17, 71]. Immune reactions to CRISPR components and delivery vectors also limit therapeutic efficacy and raise safety risks, particularly in immunocompromised patients. Pre-existing antibodies to Cas proteins or viral capsids can trigger inflammation and rapid clearance [72]. To address this, non-immunogenic Cas protein and viral capsid engineering are under development [71, 73, 74]. In addition, the long-term safety concern of CRISPR therapies remains under investigation. Risks such as delayed genotoxicity, chromosomal rearrangements, sustained expression of CRISPR molecules, and the development of resistance require long-term monitoring in clinical trials.

The complexity and high cost of producing CRISPR drugs also limit their clinical scalability, highlighting the need for efficient, scalable manufacturing platforms. Emerging strategies include Cas-expressing cell lines and stem cell-based systems, which offer advantages such as self-renewal and differentiation capacity. Another innovative strategy involves the in situ delivery of CRISPR components directly into the TME, which may bypass the need to produce and administer large numbers of edited cells [75].

While CRISPR-based cancer therapies primarily involve somatic cell editing, ethical concerns remain. There is limited longitudinal data on how edited genes behave over the course of patients’ lifetime. This raises concerns regarding the durability, long-term safety, and potential late-onset consequences, such as secondary malignancies or organ dysfunction. Given these uncertainties, ensuring truly informed consent before CRISPR therapy remains a challenge.

## Conclusion

CRISPR-based genome editing offers unprecedented advancement in targeted cancer therapy. With continued technological innovation and regulatory progress, the CRISPR system is poised to revolutionize personalized cancer therapy. Emerging promising developments, such as improved off-target detection, tumor-specific delivery systems, AI-optimized guide RNAs, non-immunogenic CRISPR variants, and novel delivery, hold great promise. Key challenges, such as off-target effects, delivery inefficiency, tumor heterogeneity, immunogenicity, and ethical concerns, must be addressed, particularly for clinical applications. Ongoing efforts to develop high-fidelity CRISPR tools, RNA-based systems, and innovative delivery platforms (e.g., Ads, LNPs, exosomes) represent promising strategies to improve safety and efficacy. Continued optimization of CRISPR technologies will be critical to transforming the future of cancer treatment.
